# Supplementary material for: Health and Safety Risk Mitigation among Artisanal and Small-Scale Gold Miners in Zimbabwe
Source: Int J Environ Res Public Health. 2022 Nov 2;19(21):14352. doi: 10.3390/ijerph192114352 (PMC9659187; doi:10.3390/ijerph192114352)
Supplement: Supplementary file 1 [file ijerph-19-14352-s001.zip › ijerph-1930423-supplementary.pdf]

Supplementary Materials

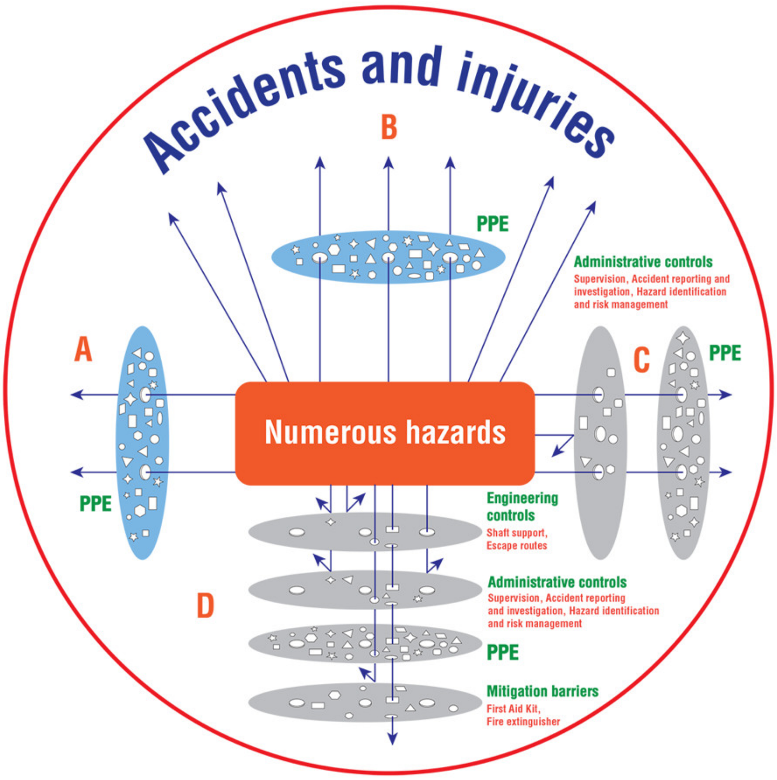

Figure S1. Numerous hazards and successive defensive layers: risk management and accident prevention in ASGM in Zimbabwe [8]. Key: high exposure to hazards: (**A**,**B**): single layer of control measures (e.g., PPE); (**C**) reduced exposure to hazards: double layer control measures (e.g., PPE, administrative controls); comprehensive protection; (**D**) successive layers of control measures (e.g., engineering controls, administrative controls, PPE, and mitigation barriers).

Table S1. Multi-causal analysis of reported risk factors contributing to the safety and health of artisanal and small-scale gold miners in Kadoma and Shurugwi in Zimbabwe in 2017 and 2020.

Immediate causes (78)	Workplace factors (246)	ASM sector-based factors (146)	Contextual factors (3)
**Behavioural (58)**	[1]***Chemicals (103)**	**Limited capital (5)**	Rain season (2)
Negative perceptions (35)	Chemical dust (34)	**Lack of PPE (37)**	Prevailing economy (1)
Preference for mercury (8)	Mercury (29)	Dust masks (7)	
Workplace violence (9)	Toxic gases (25)	Respirators (6)	
Alcohol abuse (5)	Cyanide (8)	Hearing protection (5)	
Sexual indulgence (1)	Acid & caustic soda (4)	Safety goggles (2)	
**Non-compliance (16)**	[2]***Unsafe shafts (34)**	Raincoat (1)	
Non-compliance, PPE (9)	Rock falls (16)	Gloves (2)	
[3]*Compromised waiting (3)	Collapsing mines (8)	**Inconsistent income (7)**	
[4]*Neglecting development (2)	Sub-standard shafts (3)	**Subsistence mining (2)**	
Mining survival tactics (1)	Slipping (1)	**Metal winch rope costly (2)**	
Mining of pillars (1)	**Poor sanitation (9)**	**Security guards costly (1)**	
**Equipment (3)**	[5]***High temperatures (10)**	**Insufficient food at work (8)**	
**Mistakes (1)**	**Milling (8)**	**Lack of equipment (3)**	
Forgetting to wear gloves (1)	**Biological hazards (8)**	**Compromised controls (37)**	
	Snakes (3)	[6]*Ineffective practices (10)	
	Rodents (2)	[7]*No Hg- free technology (9)	
	[8]*Ticks and mosquitoes (3)	PPE costly (individuals) (6)	
	**Confined workplaces (3)**	Substandard respirators (4)	

**Underground water (5)**	Lack of security guards (2)
**Blasting (4)**	[9]*Lack of antidote (2)
**Drilling (4)**	Lack of signage (1)
**Noise (11)**	Lack of testing equipment for gases (1)
**Poor ventilation (3)**	Lack of testing equipment for noise (1)
**Manual operations (2)**	Lack of siren for giving warning before blasting (1)
**Working underground (2)**	**Lack of training (24)**
**Poor communication (2)**	Ignorance (13)
**Electricity (1)**	Lack of education (5)
**[10]*Unsafe practices (22)**	Lack of training in mining (3)
Overworking (9)	**Informal practices (20)**
No safety talks and checks (5)	Small mining holes/pits (4)
Overcrowded shafts (2)	Lack of enforcement of regulations (5)
No provision of PPE (2)	Lack of regulations for ASM (4)
Handling explosives (1)	Gold rushes (3)
Uncontrolled blasting (1)	Unregistered miners (3)
**Incompetent people (4)**	Mining alone (1)
Untrained blasters (2)	
[11]*Inexperienced workers (2)	
**Psychosocial factors (11)**	
Stress (1)	
Oppression (1)	
Staying away from family (1)	
Violence (8)	

[1] * Chemicals (n = 3). Total for all chemicals (n = 103); [2] *Unsafe shafts (n = 6). Total for unsafe shafts (n = 34); [3] *Compromised recommended waiting time after blasting; [4] *Neglecting the initial stage of mine development; [5] *High temperatures underground shafts and summertime; [6] *Ineffective practices, e.g., (a) dry drilling without protection and taking a traditionally brewed beverage '*maheu*' or traditionally brewed beer '*chibuku*' *t*o remove inhaled dust and chemicals from the respiratory system after drilling and blasting; (b) taking drugs for courage before and after blasting; [7]*No Hg free-technology: lack of accepted and efficient mercury free-technology; [8] *Ticks (n = 2) and mosquitoes (n = 1); [9] *Lack of antidote as emergency preparedness for cyanide (highly toxic) poisoning on sites involved in cyanide leaching; [10] *Unsafe work practices; [11] *Inexperienced workers: inexperienced blasters (n = 1) and inexperienced electricians (n = 1).
